# Global Research Network Analysis of Edible Coatings and Films for Preserving Perishable Fruit Crops: Current Status and Future Directions

**DOI:** 10.3390/foods13152321

**Published:** 2024-07-24

**Authors:** Yardjouma Silue, Olaniyi Amos Fawole

**Affiliations:** 1 Postharvest and Agroprocessing Research Centre, Department of Botany and Plant Biotechnology, University of Johannesburg, P.O. Box 524, Johannesburg 2006, South Africa; siluey@uj.ac.za; 2 South African Research Chairs Initiative in Sustainable Preservation and Agroprocessing Research, Department of Botany and Plant Biotechnology, Faculty of Science, University of Johannesburg, P.O. Box 524, Johannesburg 2006, South Africa

**Keywords:** postharvest management, fruit preservation, network analysis, research agenda mapping

## Abstract

Edible coatings and films have gained substantial attention as a promising and sustainable technology for fruit preservation. This study employed a bibliometric analysis to identify core research areas, research gaps, and emerging trends, thus providing a comprehensive roadmap for future research on the use of edible coatings and films for fruit quality preservation. The study involved 428 research articles related to edible coatings and films for fruit preservation published in the Scopus database before 06 October 2023. Utilizing Vosviewer and R for network analysis, we generated network visualization maps, research performance statistics, and identified key contributors and their collaborations. The results show the evolution of this field into three distinct phases: Initial Exploration (1998–2007), Growing Interest (2008–2015), and Rapid Expansion (2016–2023). The study revealed contributions from 1713 authors, with the first article appearing in 1998. Brazil and China emerged as the most productive countries in this domain. The core research areas focus on biomaterials, functional properties, and natural substances. Identified research gaps include pilot and industrial-scale applications, the lack of a regulatory framework and safety guidelines, and the application of artificial intelligence (AI), particularly deep learning and machine learning, in this field of edible coatings and films for fruit preservation. Overall, this study offers a scientific understanding of past achievements and ongoing research needs, thus aiming to boost a broader adoption of edible coatings and films by consumers and the food industry to preserve fruit quality, thereby enhancing their societal and environmental impact.

## 1. Introduction

The increasing global human population is projected to reach 9 billion by 2050 [1], which necessitates urgent measures to address critical challenges such as food insecurity, global hunger, and malnutrition prevention. With food demand expected to double [2], food production is projected to increase by 100–110% to meet the needs of the 2050 population [3,4]. However, this increase must occur under sustainable conditions, thereby considering the environmental, social, and economic impacts of food production and distribution.

Fruits and vegetables are fundamental to the food system and are vital to human nutrition and public health. However, their production and postharvest management are confronted with challenges. These challenges include adapting to climate change, ensuring sustainable production practices, and maintaining postharvest quality and storage efficiency. According to the United Nations Food and Agriculture Organization (UN FAO) data, global fruit production reached approximately 870 million metric tons in 2018 [5], with postharvest losses in fruits and vegetables along the supply chain estimated to be between 25 and 50% [5]. This significant loss suggests the need for effective preservation strategies to ensure food security and sustainability.

The shift in consumer preferences towards fresh and minimally processed fruits has been influenced by changes in food habits and lifestyles [6]. This trend has heightened the food industry’s interest in innovative packaging solutions, particularly environmentally sustainable ones. Despite their low cost and desirable mechanical properties, traditional plastic-based films are increasingly scrutinized due to their nondegradability and environmental impact. Consequently, consumer interest in renewable, recyclable, and biodegradable materials for food packaging, such as edible coatings and films, is growing [7,8].

Edible coatings and films are composed of natural or food-grade materials like polysaccharides (chitosan, alginate, and starch), lipids (beeswax, carnauba wax, and oils), proteins (gluten and gelatin), and composites [9,10,11,12] and represent a promising technology for fruit quality preservation. These materials are physical barriers to water and gas exchange and inhibit microbial growth [13,14]. They can be applied directly to fruit surfaces or used as stand-alone structures for wrapping [15,16,17]. While polysaccharides and protein-based materials exhibit good gas barrier capacities, their hydrophilic nature limits their effectiveness as water barriers [18,19,20]. Conversely, lipid-based materials provide excellent moisture barriers but may induce off-flavors under anaerobic conditions [21].

Despite the extensive application of edible coatings and films in enhancing the shelf life of a wide range of fruits, such as apples, strawberries, tomatoes, papaya, and mangoes [22,23,24,25,26], a critical gap exists in the form of a comprehensive bibliometric analysis in this field. While existing literature reviews [27,28,29] provide valuable insights, they often rely on subjective selection criteria, thus potentially overlooking emerging trends, under-represented research areas, and interdisciplinary connections. A bibliometric analysis, in contrast, offers an objective, data-driven approach to map the intellectual landscape and conceptual evolution of edible coating and film technology in fruit preservation. It identifies key research themes, influential studies, and leading contributors, thus revealing the field’s developmental trajectory and current state [30]. Moreover, such an analysis is crucial in a rapidly evolving field where technological advancements and consumer preferences constantly reshape research priorities and application areas. By employing bibliometric analysis, this study aims to establish the underlying patterns, research gaps, and potential future directions in the use of edible coatings and films for fruit quality preservation. This approach provides a holistic view of the research landscape and guides future studies towards areas of high impact and relevance. Through this analysis, we aim to identify core research areas, essential studies, and emerging trends, thereby offering a comprehensive roadmap for future research and development in this vital field of food science. To achieve this, we pose the following research questions:

RQ1: How has scientific production evolved in edible coating and film technology for fruit preservation, and who are the key contributors?

RQ2: What are the major themes investigated in edible coatings and films for fruit preservation?

RQ3: What are the research gaps and future directions in edible coating and film technology for fruit preservation?

## 2. Results

### 2.1. An Overview of Research Performance Statistics on Edible Coatings and Films

**Figure 1:**
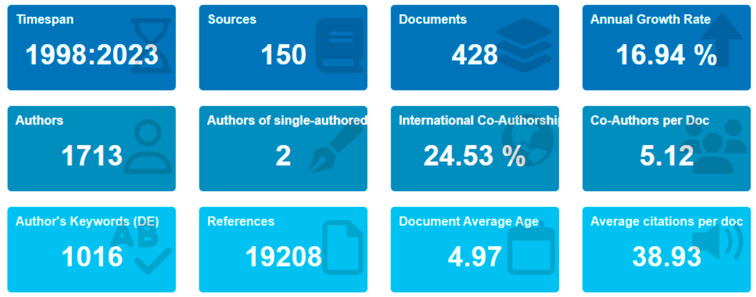
Research performance statistics on edible coatings and films.

The global scientific production in edible coatings and films (ECs/Fs) research for fruit preservation is 428 original research articles. This production concerns only the papers published in English from 1998 to 2023 in the Scopus database. They are strictly related to the development and application of edible coatings and films for fruit preservation. Furthermore, these 428 research articles have been authored by 1713 contributors across 151 sources (Figure 1). The annual growth rate shows that the research in the field of ECs/Fs is constantly increasing with time.

### 2.2. Research Trends in Edible Coatings and Films

**Figure 2:**
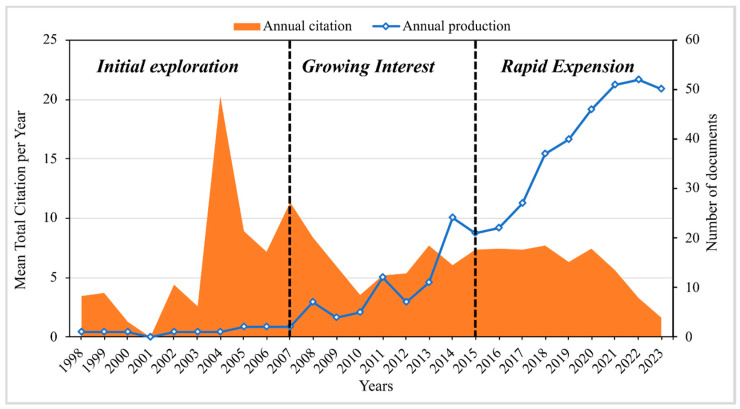
Publication trends on edible coatings and films based on the Scopus database from 1998 to 2023.

Research on edible coatings and films for fruit preservation began in 1998 with a seminal study examining the effects of starch-based coatings on strawberries under cold storage conditions [31]. This pioneering work marked the genesis of a rapidly evolving research area that, by 2023, has expanded to encompass 428 research articles. The trajectory of this field’s evolution can be delineated into three distinct phases (Figure 2), with each characterized by unique research activities and publication outputs.

The initial exploration (1998–2007) phase represents the infancy of the field, with annual publications ranging from one to two articles. The foundational research [31] laid the groundwork for future investigations, thus establishing a nascent pace for the field’s growth. During this period, research primarily focused on understanding the basic principles and potential applications of edible coatings and films for fruit preservation.

The growing interest (2008–2015) phase saw a gradual increase in research output, with annual publications fluctuating between 7 and 24 articles. This era reflects the growing recognition of the potential benefits of edible coatings and films, thereby highlighted by a steady rise in academic and industrial attention towards sustainable postharvest solutions. The research during this period began to diversify, thus exploring various materials and methods to enhance the functionality and effectiveness of edible coatings and films in preserving fruit quality. The rapid expansion (2016–2023) phase has been marked by a pronounced surge in scientific contributions, thus driven by the increasing global demand for sustainable food preservation methods and the influence of the United Nations Sustainable Development Goals (SDGs) introduced in 2015 for the promotion of SDG 12 (responsible consumption and production); this period witnessed a significant increase in research articles, from 31 funded publications between 2004 and 2014 to 195 articles from 2015 to 2023. This surge highlights the field’s alignment with global sustainability objectives and its critical role within food science and technology.

### 2.3. Geographic Distribution of Research

**Figure 3:**
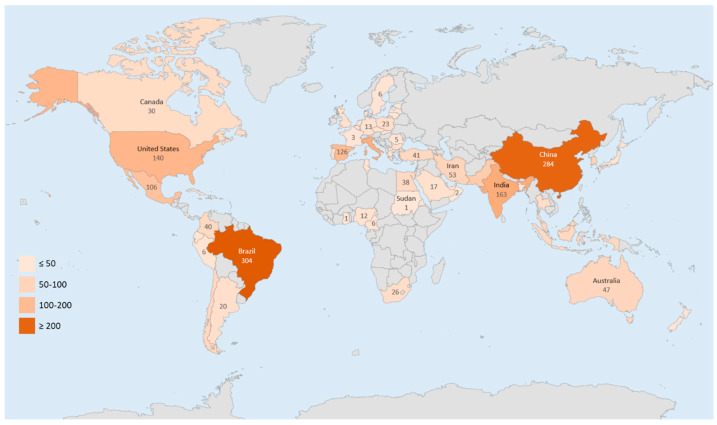
Scientific production map in the field of edible coatings and films for fruit preservation.

The global research landscape in edible coatings and films is represented by contributions from 56 countries, which are illustrating the field’s widespread relevance and applicability. The geographic distribution of research is categorized based on publication output, ranging from emerging contributors with fewer than ten papers to leading contributors with over a hundred publications. Notably, Brazil and China have emerged as the most prolific countries in this domain, which is attributable to their significant roles in fruit production, processing, and exportation. They are followed by India, Italy, the USA, Spain, and Mexico, thus indicating a strong correlation between a country’s fruit production capacity and its research output in edible coatings and films [32]. The scientific production map (Figure 3) visually encapsulates the global distribution of research efforts. Asia, led by China and India, is at the forefront, thereby accounting for over half of the region’s publications. Europe, South America, and North America follow in terms of research contributions, with Africa recording the least activity. The absence of developing countries, particularly in Africa, on the production map can be associated with a lack of laboratory equipment and low funding for research in these regions. Moreover, the interactive nature of research in edible coatings and films is evident as productive countries collaborate globally. These international collaborations further emphasize the importance of this field in addressing global challenges related to fruit preservation and sustainable food practices.

### 2.4. Global Collaboration Network in EC/F Research

**Figure 4:**
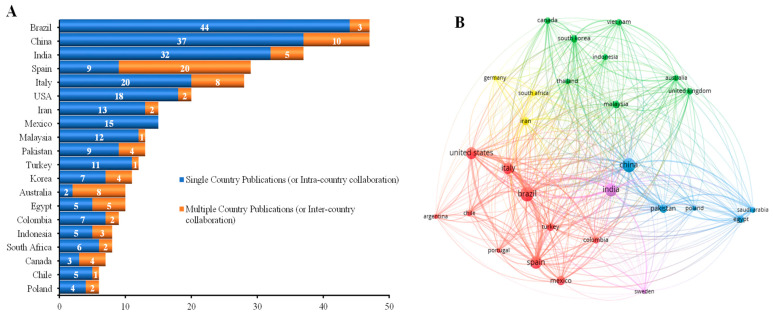
Global collaboration including countries with a minimum number of 5 documents of a country (**A**) and network visualization map of countries through bibliographic coupling analysis of retrieved documents on ECs/Fs research in Scopus (1998–2023) (**B**). The country is represented by a node, and the size of the node indicates the number of papers published by the institution/author in the country. The line thickness between two countries means linked countries occurred together, while the closeness represents the frequency of this co-occurrence. Each colour in (**B**) represents a distinct cluster, designating a group of countries that have strong collaborative research networks. The clusters are determined based on documents-weights.

The research landscape of edible coatings and films (ECs/Fs) is marked by significant contributions from diverse countries, thus underscored by a dynamic global collaboration network. This section delves into country-specific contributions to the field and examines the intricate web of global collaborations that underpin ECs/Fs research (Figure 4A).

The global collaboration network in ECs/Fs research is characterized by multifaceted partnerships spanning different continents, thus facilitated by shared research interests in enhancing food preservation sustainably and addressing postharvest losses. A critical metric in this context is the international collaboration ratio, which measures the proportion of a country’s research output produced in collaboration with international partners. A high international collaboration ratio indicates a strong engagement in cross-border research efforts. Countries such as Australia, Spain, Canada, and Egypt exhibit high international collaboration ratios, thus signifying their active participation in global research projects. For instance, Australia’s leading position in international collaborations can be attributed to its unique expertise in niche fruit markets and the necessity to meet rigorous export quality standards. These collaborations often address common challenges, such as extending the shelf life of tropical fruits, developing biodegradable packaging solutions, and integrating natural antimicrobials and antioxidants into edible films. Collaboration networks extend beyond bilateral partnerships to encompass multi-country consortia driven by global initiatives and research grants that foster interdisciplinary and crossborder research efforts. This extensive network of collaborations enhances the exchange of knowledge, resources, and technological innovations across countries. Bibliographic coupling analysis provides insights into the influence of different countries in ECs/Fs research, thereby highlighting Spain, Brazil, China, India, and the United States as central nodes in the collaboration network (Figure 4B). These countries contribute significantly to the field and play pivotal roles in shaping the global research agenda through their extensive international collaborations. The analysis of country-specific contributions and global collaboration networks reveals a vibrant and interconnected research community dedicated to advancing ECs/Fs technologies. The leadership of countries like Brazil and China and the extensive international collaborations led by Australia and Spain underscores the global commitment to enhancing food preservation through innovative and sustainable technologies. As the field continues to evolve, these collaborations will be crucial in addressing the complex challenges of food security, sustainability, and climate change impacts on agriculture.

### 2.5. Network Visualisation of Academic Journals within Edible Coating and Film Research

The network analysis of ECs/Fs research highlights the central role of leading journals in fostering the dissemination and crosspollination of knowledge within the field. These journals serve as repositories of cutting-edge research and hubs connecting diverse strands of inquiry, thus contributing to the advancement of edible coatings and films as sustainable solutions for fruit preservation. The interconnectivity among them underscores the collaborative and interdisciplinary nature of ECs/Fs research, thus reflecting its importance in addressing global challenges related to food security, sustainability, and environmental conservation.

Leading journals in the EC/F research landscape serve as primary platforms for the publication of original, innovative findings and theoretical advancements. As presented in Table S1, “Postharvest Biology and Technology”, one of the top-three productive sources appears as a most impactful publication venue, thus reflecting its focus on the postharvest handling of perishable products and its relevance to ECs/Fs research (Table S1). Similarly, “LWT—Food Science and Technology” also plays a significant role, given its emphasis on the processing, preservation, and distribution of food. “International Journal of Biological Macromolecules”, the most productive in this field, is prominent for its broad coverage of the chemical and biological aspects of materials used in food packaging technologies for food safety. These journals, among others, constitute the core of the ECs/Fs research dissemination network, thus underscoring their importance in advancing the field.

**Figure 5:**
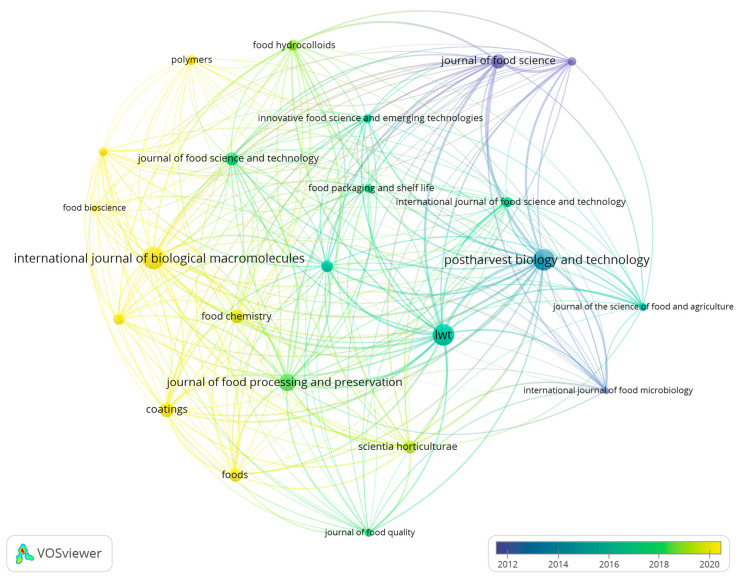
Network visualization map of academic journals in the field of edible coatings and films for fruit preservation (1998–2023) in the Scopus database. Each node in a network represents a journal, and the size of the node indicates the number of publications in the journal. The bigger node signals a high number of publications. The edge (link) between the two journals represents the bibliographic coupling. The color scale shows the average publication year of each keyword, where the color coding for each keyword is defined by the average publication year of all publications with the keyword.

Identifying leading journals and analyzing their network highlights how research findings are interconnected and shared across the scientific community, thus facilitating the flow of knowledge and fostering collaborative research endeavors. The network analysis of ECs/Fs research journals illustrates the interconnected nature of scientific publishing in this area (Figure 5). The most productive journals, such as the “International Journal of Biological Macromolecules”, “Postharvest Biology and Technology”, and “LWT” frequently crossreference each other and all other journals with more than five documents, thus indicating a strong thematic overlap in the topics of postharvest management, edible materials, biopolymers, and their applications in food science. This interconnection is further evidenced by the frequent citation of articles across these journals, thus highlighting the collaborative nature of ECs/Fs research.

The network analysis also sheds light on the role of interdisciplinary journals in bridging diverse research areas. An overlay visualization map of journals (more than five documents) generated from bibliometric data reveals the recent trends in ECs/Fs research (Figure 5). The items’ colors are defined based on their average publication per year, thus forming different clusters of journals with similar annual growth rates. The current most productive journals are colored in yellow and led by the “International Journal of Biological Macromolecules”. Journals in this cluster tend to focus on specific research themes such as promoting natural materials (macromolecules, antimicrobials, antioxidants, and plasticizers), food safety, and techniques and methods used for food packaging, characterization, production, and processing, especially their sustainability. This visualization aids in identifying emerging trends and gaps in the literature, thus guiding future research directions.

The network analysis of ECs/Fs research, therefore, highlights the central role of leading journals in fostering the dissemination and crosspollination of knowledge within the field. These journals serve as repositories of cutting-edge research and as hubs that connect diverse strands of inquiry, thus contributing to the advancement of edible coatings and films as sustainable solutions for fruit preservation. The interconnectivity observed among them underscores the collaborative and interdisciplinary nature of ECs/Fs research, thus reflecting its importance in addressing global challenges related to food security, sustainability, and environmental conservation.

### 2.6. Dominant Themes in Edible Coatings and Films Research

#### 2.6.1. Development of Novel Materials

**Figure 6:**
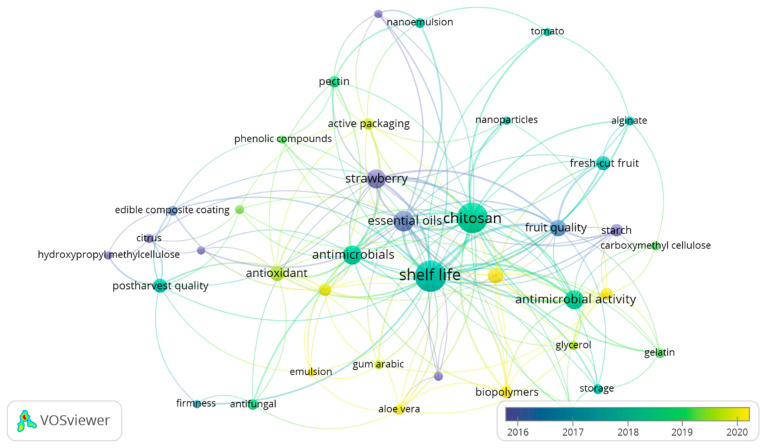
Overlay visualization map exhibiting the most keywords in edible coatings and films for fruit preservation from 2016–2020 in the Scopus database. Each node represents a keyword, and the size of the node designates the occurrence of the keyword. The bigger node signals a high occurrence of the keyword. The link connecting two keywords indicates the number of times they co-occur. The color scale shows the average publication year of each keyword, where the color coding for each keyword is defined by the average publication year of all publications with the keyword.

Central to the advancement of edible coatings and films is the innovation in material science, thus focusing on biodegradable and natural substances. Polysaccharides, proteins, lipids, and composite materials have been extensively studied for their potential in food preservation. Among polysaccharides, chitosan, a biodegradable polymer derived from chitin, is noted for its antimicrobial properties and has been widely researched for its efficacy in extending the shelf life of fruits [33]. Alginate, another polysaccharide, is valued for its ability to form effective barriers against moisture and oxygen, which is essential in minimizing fruit degradation [34]. Proteins such as gelatin and wheat gluten have been explored for their film-forming capabilities, thus contributing to the development of coatings that provide gas and vapor barriers [35,36]. Lipids, including beeswax and carnauba wax, offer hydrophobic properties that significantly reduce water loss, a critical factor in maintaining fruit freshness [37,38]. Composite materials that combine these biopolymers aim to harness the synergistic effects of their protective properties, thereby offering enhanced performance in fruit preservation [12,39]. Figure 6 presents the chronological introduction of these materials in edible coatings and film production for fruit preservation. Thus, *Aloe vera* and gum Arabic have been recently introduced as materials for edible coatings. Additionally, the introduction of these new materials is also correlated with the use of antioxidants and the study of the antioxidant activity of edible coatings and films.

#### 2.6.2. Enhancing ECs/Fs Efficacy for Shelf Life and Quality

The application of edible coatings and films is primarily aimed at extending the shelf life of fruits by modulating respiration rates, decreasing moisture loss, and inhibiting microbial growth. Studies have shown that chitosan-based coatings effectively delay ripening and reduce decay in strawberries and raspberries, thus prolonging their freshness and marketability [40]. Alginate-based films have been used to preserve the firmness and color of fresh-cut apples, thus underscoring their utility in maintaining the quality of minimally processed fruits [34]. Furthermore, any of these materials used individually do not allow for controlling water loss, fruit respiration, ethylene production, and microbial growth. Therefore, to enhance the efficacy of edible coatings and films, research has been oriented towards the combination of materials and the incorporation of bioactive compounds (antimicrobials and antioxidants) (Figure 6). However, this requires specific standards related to sustainability and safety.

#### 2.6.3. Sustainable Solutions to Postharvest Challenges

With growing environmental concerns over synthetic packaging materials, edible coatings and films have emerged as eco-friendly alternatives. These materials, sourced from renewable resources, align with the global push towards sustainability. The integration of natural substances such as essential oils and plant extracts into these coatings not only enhances their antimicrobial and antioxidant capabilities but also meets consumer demand for natural and sustainable food preservation methods [34,41]. This approach ensures the safety and quality of the preserved fruits and promotes environmental sustainability by reducing the reliance on nonbiodegradable packaging.

### 2.7. Material Innovations for Edible Coatings and Films

Research in edible coatings and films has seen substantial material innovation, which is pivotal for enhancing the postharvest quality and shelf life of fruits. These advancements are central to the development of sustainable packaging solutions, thus addressing both food preservation needs and environmental concerns. Emerging materials such as biopolymers (e.g., chitosan and alginate), nanomaterials (e.g., nanoemulsions and nanofibers), and composites have been integrated with natural antioxidants like ascorbic acid, tocopherols, and plant extracts to improve their functional properties. These innovations enhance barrier properties, antimicrobial effects, and the controlled release of active compounds, thus significantly mitigating oxidative stress and microbial spoilage. The synergy between these advanced materials and antioxidants extends the shelf life of fruits and contributes to the creation of eco-friendly and effective food preservation solutions.

#### 2.7.1. An Overview of Materials Used in Edible Coatings and Films

##### Polysaccharides

Polysaccharide-based materials, such as chitosan, alginate, and starch, are widely utilized due to their biocompatibility, biodegradability, improved mechanical properties, and effective barrier properties against oxygen. Chitosan, derived from chitin, is renowned for its antimicrobial properties, thus making it a preferred choice for extending the shelf life of perishable crops [42]. Alginate, sourced from brown seaweed, forms hydrophilic films that effectively prevent hydration and nutritional quality loss in fruits [25]. However, polysaccharide-based coatings and films are limited by their poor water vapor barrier properties, while lipid-based coatings provide high water vapor barrier characteristics.

##### Lipids

Lipid-based coatings, including beeswax and carnauba wax, are used for their hydrophobic nature, thus providing a moisture barrier and significantly reducing water loss from the fruit surface. These materials are often combined with polysaccharides or proteins to improve the mechanical and barrier properties of composite film [41].

##### Proteins

Proteins, like gelatin, casein, and whey protein isolate, are used for their excellent film-forming abilities and nutritional benefits. These materials create a breathable layer over the fruit surface, which helps in gas exchange and retains the fruit’s freshness while adding nutritional value [12].

##### Composite Materials

The synergy between different material types has led to the development of composite coatings and films. These composites harness the unique properties of each constituent material, thus offering enhanced barrier properties, mechanical strength, and functional benefits such as improved antimicrobial activity [43].

**Figure 7:**
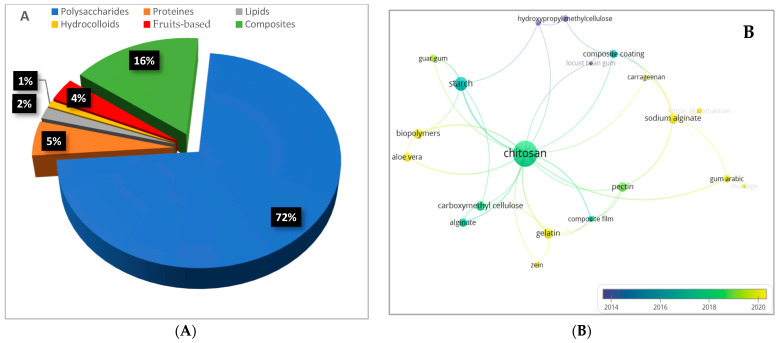
Proportion of biomaterials studied within the retrieved documents from the Scopus database on edible coatings and films (**A**) and overlay visualization map of biomaterials cited in more than five documents as keywords by authors in the field of edible coatings and films for fruit preservation (1998–2023) in the Scopus database (**B**). Each node represents a keyword, and the size of the node designates the occurrence of the keyword. The bigger node implies a high occurrence of the keyword. The link between the two keywords indicates that they co-occur together. The color scale shows the average publication year of each keyword, where the color coding for each keyword is defined by the average publication year of all publications with the keyword.

Based on the retrieved and screened documents (428) from the Scopus database, polysaccharides are the most researched materials for edible coatings and films for fruit preservation, thus constituting 72% of the biomaterials studied within the retrieved documents (Figure 7A). This equates to 136 documents, making it the most used material (Table S2). Lipids constitute only 2% of the biomaterials studied (Figure 7A), with gelatin being the most-used protein for ECs/Fs, as shown in the research trend analysis over the last decade in the field in Figure 7B. Recent material innovations also include the use of Aloe vera, gum Arabic, and various mucilage, which are being explored for their potential in edible coatings and films (Figure 7B). The chronological introduction of these materials highlights the ongoing advancements and trends in the development of edible coatings and films (Figure 7B). These innovations contribute significantly to the sustainability of food packaging, thus promoting the use of renewable resources and reducing the reliance on nonbiodegradable materials.

#### 2.7.2. Technological Advancements for Enhanced Functionality of ECs/Fs

##### Nanotechnology

The integration of nanotechnology in edible coatings and films introduces nanoparticles that can provide targeted delivery of nutrients or antimicrobials, improve mechanical strength, and enhance barrier properties. For instance, the nanoencapsulation of essential oils within chitosan-based coatings has shown increased efficacy in antimicrobial activity against pathogenic bacteria [44]. The nanoscale materials include nanoparticles, nanocomposites, and nanofibers. The application of nanomaterials allows for fine control over biomaterial properties, reduces waste, and ensures food safety. However, nanotechnology-based packaging requires a deep examination of its effect in terms of long-term safety and environmental impacts [45].

##### Active Packaging

Active packaging technologies incorporate bioactive compounds directly into the edible coating/film matrix. These compounds, including antioxidants and antimicrobials, actively interact with the fruit to extend shelf life and enhance safety. Essential oils, plant extracts, and natural preservatives are commonly used, thus offering a dual function of preservation and sensory enhancement [34].

##### Smart Packaging

Smart edible coatings and films can respond to environmental changes or fruit ripening stages. These smart materials can adjust their permeability or release bioactive compounds based on the fruit’s needs, thus ensuring optimal preservation conditions throughout the supply chain [46,47].

##### Bioprinting

Advanced manufacturing techniques such as 3D bioprinting allow for the precise application and customization of edible coatings and films. This technology enables the development of multilayered coatings with tailored properties for specific fruit types, thereby optimizing protection and extending shelf life [48].

Given the above-mentioned technological advancements, it is clear that innovations in materials and technology for edible coatings and films are at the forefront of addressing the dual challenges of food preservation and environmental sustainability. By harnessing the potential of polysaccharides, proteins, lipids, and composite materials and by leveraging advancements such as nanotechnology and smart packaging, the field is poised to offer effective and adaptable solutions for the postharvest treatment of fruits.

#### 2.7.3. Functional Properties of Edible Coatings and Films

The functional properties of edible coatings and films, including barrier, mechanical, and bioactive properties, play a critical role in their effectiveness in extending the shelf life of fruits. These properties contribute to maintaining the postharvest quality of fruits and offer new dimensions to sustainable food preservation practices.

##### Barrier Properties

Edible coatings and films serve as selective barriers against oxygen, carbon dioxide, moisture vapor, and ethylene, thereby modulating the internal atmosphere around the fruit. This can significantly slow the respiration rate, delay ripening and senescence, and reduce moisture loss, thus prolonging shelf life. For instance, gelatin/carboxymethyl cellulose-based composite films have been shown to effectively reduce water vapor permeability, which is crucial for minimizing dehydration in fruits such as berries [49]. Chitosan coatings, due to their semipermeable nature, have been reported to maintain the internal gas composition favourable for extending the shelf life of bananas and avocados by inhibiting ethylene production [33].

##### Mechanical Properties

The mechanical strength, elasticity, and flexibility of edible coatings and films are essential for ensuring the physical integrity of the coated fruit during handling and storage. These properties depend on the polymer matrix’s composition and structure and can be tailored to match the application needs. For example, composite films incorporating both polysaccharides and proteins have demonstrated improved tensile strength and flexibility, thus making them suitable for delicate fruits like berries [50]. The incorporation of plasticizers, such as glycerol, can further enhance the flexibility and extensibility of these films, thus allowing for better conformity to the fruit’s surface.

##### Bioactive Properties

Edible coatings and films can be fortified with natural antimicrobials, antioxidants, and nutrients to provide additional functional benefits. These bioactive compounds can inhibit microbial growth, reduce oxidative stress, and supply essential nutrients, thereby improving the safety, nutritional value, and sensory attributes of the fruit. A notable example is the use of *Aloe vera*-based edible coatings enriched with grapefruit seed extract on grapes, which significantly reduced fungal infections and preserved the fruit’s quality during cold storage [51]. Another example is the use of chitosan films incorporated with green tea extract, which exhibited improved mechanical properties and water vapor barrier properties and enhanced polyphenolic content and antioxidant activity of the films [52].

##### Fruit Shelf Life Extension

**Figure 8:**
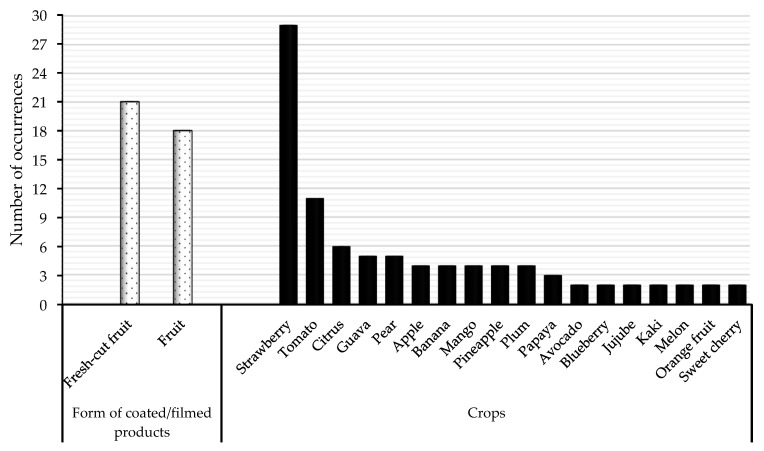
Crops commonly studied in edible coatings and film research based on the retrieved documents (428) from Scopus (1998–2023).

The literature contains several success stories where edible coatings and films have markedly extended the shelf life of various fruits. Based on 428 research articles from the Scopus database, the preservation of strawberries is the most researched using edible coatings and films (Figure 8). Moreover, fresh-cut fruits are more studied than whole fruits, and edible coatings and films have been widely applied to many tropical and temperate fruits. One such example is the application of alginate film incorporated with ZnO-NPs on tomatoes, which resulted in a significant delay in ripening and a reduction in postharvest decay, thus effectively extending the fruit’s shelf life up to 16 days without any defects [53]. Similarly, beeswax-based edible coatings have been found to preserve the texture and colour of plums for an extended period, thereby maintaining their marketability and reducing waste [54].

Overall, the functional properties of edible coatings and films, including their barrier, mechanical, and bioactive attributes, are fundamental in enhancing fruit preservation. Through technological advancements and the integration of bioactive compounds, these edible materials offer promising solutions for extending the shelf life of fruits, thus contributing to food sustainability and reducing postharvest losses. The success stories highlighted in the literature highlight the potential of these innovations in meeting the challenges of global food security and preservation.

#### 2.7.4. Incorporation of Natural Substances

The incorporation of natural substances into edible coatings and films represents an important advancement in postharvest preservation, primarily due to their antimicrobial and antioxidant properties. These natural additives enhance the protective capabilities of coatings and films and align with the consumer demand for more natural and safe food preservation methods.

##### Natural Antimicrobials

Natural antimicrobials, including essential oils, plant extracts, and bioactive peptides, have been widely researched for their potential to inhibit or prevent the growth of pathogenic and spoilage microorganisms on fruits. Essential oils such as thyme, oregano, and cinnamon have shown significant antimicrobial activity when incorporated into edible coatings and films, thus effectively reducing microbial load and extending the shelf life of various fruits [55,56,57]. Furthermore, chitosan films enriched with *Thymus capitatus* oil have demonstrated a remarkable ability to inhibit fungal growth on strawberries, thus maintaining their freshness and reducing spoilage [58]. Similarly, grapefruit seed extract, used as an antimicrobial agent in chitosan-based coatings, has been effective in controlling the growth of Salmonella on cherry tomatoes, thus ensuring safety during storage [59].

##### Natural Antioxidants

Antioxidants play a crucial role in preventing oxidative stress and enzymatic browning in fruits, which are factors that significantly affect their quality and shelf life. Natural antioxidants, including vitamin E, ascorbic acid, and plant polyphenols, have been successfully incorporated into edible coatings and films to enhance their protective capabilities. For example, alginate coatings containing ascorbic acid and citric acid have been used to preserve the antioxidant capacity of fresh-cut apples, thus preventing browning and maintaining their visual appeal [60]. The incorporation of green tea extract into chitosan coatings has also been shown to provide antioxidant protection for kiwifruit, thus extending its shelf life by delaying the onset of senescence [61]. Furthermore, some essential oils are often used as antioxidants in edible coatings and films [62,63,64].

**Figure 9:**
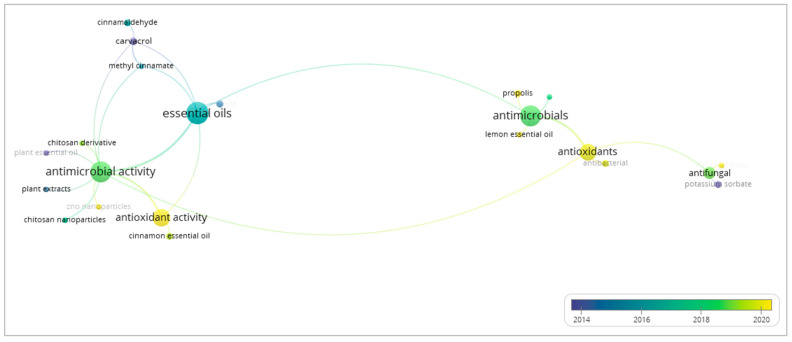
Overlay visualization map of natural substances cited as keywords by authors in papers on edible coatings and films for fruit preservation published from 1998 to 2023 in the Scopus database. Each node represents a keyword, and the size of the node designates the occurrence of the keyword. The bigger node implies a high occurrence of the keyword. The link between the two keywords indicates that they co-occur together. The color scale shows the average publication year of each keyword, where the color coding for each keyword is defined by the average publication year of all publications with the keyword.

As indicated in the network analysis of natural substances added into edible coatings and films in Figure 9, essential oils are primarily used for their antimicrobial and antioxidant properties. In addition, the overlay visualization suggests that edible ECs/Fs research has been directed towards the study of the antioxidant properties of integrated natural substances. For instance, numerous studies have investigated the antioxidant activity of various essential oils such as lemon essential oil, cinnamon essential oil, and clove essential oil [62,63,64].

### 2.8. Emerging Trends, Research Gaps, and Future Directions

As the global emphasis on sustainable food preservation intensifies, research on edible coatings and films (ECs/Fs) stands at the precipice of significant scientific evolution. Despite remarkable strides in this field, particularly in extending the shelf life and enhancing the quality of perishable fruits, several crucial research gaps remain. These gaps thus present opportunities for groundbreaking advancements that could redefine sustainable food preservation approaches.

#### 2.8.1. Emerging Trends

Emerging trends in the field, as identified in the literature, suggest a promising trajectory towards the integration of nanotechnology, thus offering novel avenues to augment the barrier, mechanical, and bioactive properties of ECs/Fs [65,66]. The advent of smart and active packaging solutions, which are capable of monitoring fruit quality and interacting with the environment, ushers in a new era of intelligent food preservation systems. The exploration of natural and upcycled materials for EC/F fabrication aligns with the global mandate for sustainability and waste reduction. Utilizing agroindustrial by-products not only mitigates waste but also adds value to otherwise discarded resources, thus fostering a circular economy. As reported by Mahmud et al. [45], the food packaging market was projected to be valued at USD 346.5 billion in 2021, while the bioplastics market is likely predicted to reach USD 2.87 million in 2025, which is a 36% increase from 2020. The concept of multifunctional coatings, which amalgamate antimicrobial, antioxidant, and nutritional enhancements, represents a holistic approach to food preservation. Such innovations could significantly impact public health by improving the nutritional value and safety of fruits.

Lastly, the intersection of ECs/Fs technologies with personalized nutrition and functional foods opens vistas for tailored health solutions. This aligns with the increasing consumer preference towards health-centric diets, thus positioning ECs/Fs as a vehicle for delivering targeted health benefits.

#### 2.8.2. Research Gaps

While the aforementioned trends are supported by bibliographic data, the identification of research gaps and future directions reflects our expert analysis and interpretation. Based on keyword analysis and the most-cited articles (Table S3), extracted articles on edible coatings and films for fruit preservation are widely investigated. Material-based ECs/Fs and natural substances [12,39,67,68,69,70,71] and their properties include antimicrobial and antioxidant activities, water and gas barriers, and mechanical properties [72,73,74].

However, one of the foremost areas necessitating rigorous exploration is the comprehensive assessment of the biodegradability and environmental impacts of ECs/Fs. While these innovations represent a shift towards greener alternatives, the dearth of lifecycle analyses poses questions about their overall sustainability and environmental friendliness. Additionally, there is a lack of elucidation of the ecological footprint of these materials, thus ensuring that their benefits surpass those of traditional packaging solutions. Furthermore, the regulatory landscape for edible coatings and films (ECs/Fs) remains fragmented, with a conspicuous absence of harmonized standards and safety evaluations, especially for materials at the frontier of food contact substance innovation. This gap highlights the need for a concerted effort towards the establishment of global regulatory frameworks that would facilitate the integration of ECs/Fs into the mainstream, thus sustained by consumer confidence and statutory compliance.

Moreover, the economic viability and scalability of ECs/Fs technologies underpin their potential for widespread adoption. Current research often circumvents the pragmatic challenges associated with commercial-scale production, including cost-effectiveness and resource optimization. Addressing these issues is paramount to transcending the gap between laboratory success stories and real-world applications. Consumer perceptions and the sensory impact of ECs/Fs on fruits also warrant deeper investigation. Insights into consumer acceptance and the influence of ECs/Fs on taste, texture, and appearance can guide the refinement of these technologies, thus tailoring them to meet market demands and enhance consumer appeal.

#### 2.8.3. Future Directions

The future of ECs/Fs in the industry is poised for growth, thus driven by ongoing research and innovation aimed at enhancing the functionality, cost-effectiveness, and consumer acceptance of these technologies. Key areas for future development include the integration of smart functionalities into ECs/Fs, thus enabling the real-time monitoring of food quality and safety and the exploration of novel materials and formulations that offer improved barrier properties, biodegradability, and nutritional benefits. Additionally, more focus is required in scaling up ECs/Fs technologies for industrial applications and navigating the regulatory landscape for widespread adoption. Furthermore, the utilization of natural and biodegradable materials in ECs/Fs deserves more attention, as this can open new market opportunities in the growing sector of sustainable food packaging, thereby attracting eco-conscious consumers and meeting the regulatory requirements for environmental stewardship. Lastly, the toxicity of these natural substances requires an accurate investigation to ensure their innocuity for consumers. In conclusion, to reach the broader adoption of edible coatings and films as an efficient and sustainable solution for preserving fruit quality, there are still a lot of challenges to face and many gaps to be filled.

## 3. Methodology

The methodology of this study was structured to ensure a comprehensive systematic and bibliometric analysis. The initial step involved defining the aim and scope of the study. The scope was deliberately broad to encompass all research aspects pertinent to edible coatings and films for fruit preservation. This included, but was not limited to, the materials used, their effectiveness in preservation, cost considerations, scalability of applications, safety profiles, sustainability impacts, and various application techniques. The next critical step was the selection of an appropriate database for data collection. The Scopus database (https://www.scopus.com/, accessed on 6 October 2023) was chosen for its extensive and comprehensive coverage of scientific publications. Scopus is renowned for its curated bibliometric data, which are crucial for a study of this nature [75]. Its wide-ranging collection of literature encompasses diverse fields, thus making it an ideal source for multidisciplinary topics like edible coatings and films.

### 3.1. Data Collection

Data collection for this study was conducted following the Preferred Reporting Items for Systematic Reviews and Meta-Analyses (PRISMA) guidelines, as detailed by Moher et al. [76]. The PRISMA method involves four steps: Identification, Screening, Eligibility, and Inclusion.

#### 3.1.1. Identification

Data collection commenced on 06 October 2023, from the Scopus database, known for its extensive academic publication coverage. The search strategy utilized specific search strings within the “Title-Abstract-Keywords” fields. These strings contained a combination of Boolean operators (OR and AND), truncation signs (*), and quotation marks (“…”) to ensure comprehensive and relevant retrieval. The search strings are presented in the supplementary document.

#### 3.1.2. Inclusion and Exclusion Criteria

**Figure 10:**
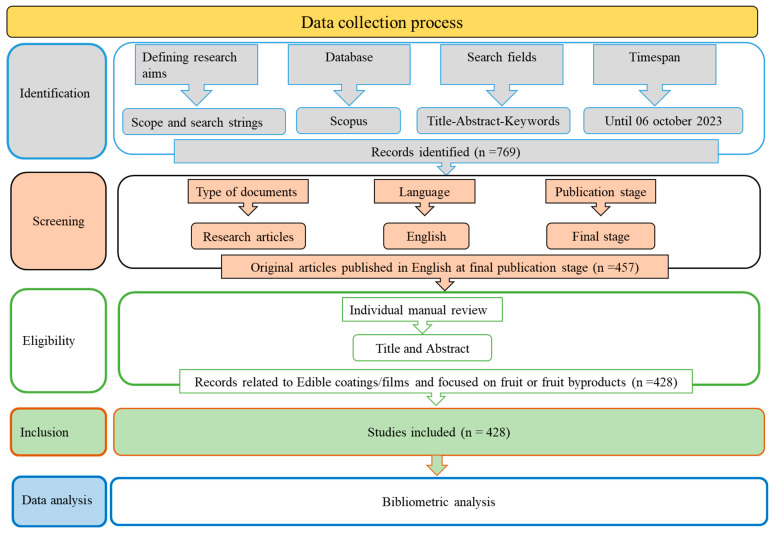
Flow diagram of our search strategy inspired by PRISMA.

The search yielded a broad spectrum of articles, including only those in English and published before the data retrieval date. This step was essential to ensure the analysis was based on the latest and most relevant literature. A manual review of the identified records was then conducted to exclude documents not specifically related to edible coatings, films, or those not focused on fruit or fresh-cut fruit. The final step involved exporting the records meeting our inclusion criteria in .csv format for bibliometric analysis. This process was crucial for building a robust dataset for our study. Figure 10 presents a flow diagram of the search strategy inspired by the PRISMA model to visually represent the data collection process. From a total of 769 retrieved documents, only 428 publications were considered eligible for further analysis.

### 3.2. Analytical Tools and Preprocessing

For the bibliometric analysis and text mining of edible coatings and films for fruit preservation, Vosviewer v. 1.6.19 was utilized for network visualization and the Bibliometrix v. 4.1.3 package in R Studio for comprehensive bibliometric analysis. Given the limitations of these tools in data preprocessing [77], Microsoft Excel v. 2308 and OpenRefine v. 3.7.5 (http://openrefine.org/, accessed on 29 September 2023) were employed for data cleaning to ensure the dataset’s accuracy, consistency, and reliability. This preprocessing stage involved merging duplicate records, harmonizing variations in author names and affiliations, standardizing synonyms and keyword variations (e.g., “postharvest” vs. “post-harvest”), and removing irrelevant words and singular/plural discrepancies. A manual review followed to guarantee the database’s quality for subsequent analysis.

### 3.3. Bibliometric Analysis and Visualisation

To address RQ1, scientific production in the edible coatings and films was analysed, thus focusing on key contributors (authors, institutions, countries) using productivity and impact indicators (number of documents, citations, h-index, m-index) as outlined by Gonzales-Malca et al. [78]. Relationships among these contributors were explored through science mapping analysis [79], which was enhanced by network visualization. Keyword frequency and co-occurrence, along with thematic evolution analysis, illuminated research gaps and future directions (RQ2 and RQ3). Factorial analysis (thematic clustering) was applied to reduce the subjectivity of interpretation and ensure objective interpretation.

## 4. Limitations and Perspectives

Although our study presents major and relevant information related to research on edible coatings and films for fruit preservation, it has some limitations. The bibliometric analysis is focused on documents retrieved only from the Scopus database, thus excluding many other databases such as Web of Science, Google Scholar, MDPI, Dimensions, and Lens. While this might appear to be a limitation, it is important to note that Scopus is considered the most authentic database of scholarly publications. It includes high-quality curated content selected by an external content selection and advisory board of subject experts, thus covering publications from other reputable sources, including MDPI journals. Thus, numerous publications could not be considered and might slightly change the results. The second weakness of the study is the limitation to only original articles and the English language, while other document types such as conference proceedings, theses (PhD and MSc), books, and book chapters can also bring meaningful information to this literature survey. The exclusive use of English can skew the results in favor of English-speaking contributors (countries). Furthermore, while edible coatings and films are applied to fruits and vegetables, our study focuses solely on fruit. This focus was chosen to provide a more detailed and specific analysis within the context of fruit preservation, which is a significant area of research due to the perishability and economic importance of fruits. However, this focus does present a limitation, as it excludes relevant research on vegetables. Future research should address these limitations to remove all potential biases in the results. Despite these limitations, the relevance and quality of this study remain intact, thus offering valuable insights into the current state and future directions of edible coatings and films for fruit preservation.

## 5. Conclusions

This study analysed the bibliometric information of 428 original articles related to edible coatings and films in preserving fruit quality, which were registered in the Scopus database from 1998 to 06 October 2023. It also included a systematic review of papers. This is the first study combining bibliometric analysis and systematic review of the topic, which shows a comprehensive overview, including research contributors, trends through the co-occurrence analysis of keywords, and research gaps and agenda. This study shows that Brazil and China are the most productive countries for edible coatings and film research publications. In addition, 84% of the 1713 authors contributing to research on the topic have only one paper, and the prolific author has 14 papers. Furthermore, the results revealed that materials, functional properties, and natural substances are the focus of research on edible coatings and films for fruit preservation. Thus, the lack of regulatory framework, safety guidelines, and studies of pilot and industrial-scale applications have constituted the research gaps in the field. Therefore, this bibliometric analysis offers many opportunities to understand what ongoing research should be conducted to have a broader adoption of edible coatings and films for preserving fruit quality by consumers and food industries and increase their impact on society and the environment.

## Data Availability

The original contributions presented in the study are included in the article/Supplementary Materials, further inquiries can be directed to the corresponding author.

## Supplementary Materials

The following supporting information can be downloaded at https://www.mdpi.com/article/10.3390/foods13152321/s1, Table S1: Most impactful academic journals for edible coatings and films for preserving fruit from 1998 to 2023 in Scopus database. Table S2: Biomaterials used for forming edible coatings and films based on the documents published in the Scopus database before 06 October 2023. Table S3: List of documents with ≥100 citations.
