# Supplementary material for: Global Research Network Analysis of Edible Coatings and Films for Preserving Perishable Fruit Crops: Current Status and Future Directions
Source: Foods. 2024 Jul 24;13(15):2321. doi: 10.3390/foods13152321 (PMC11311519; doi:10.3390/foods13152321)
Supplement: Supplementary file 1 [file foods-13-02321-s001.zip › foods-3094983-supplementary.pdf]

Search strings

[("edible coating" OR "edible film*") AND "fruit*" AND ("hydrocolloid coatings" OR "protein-based coatings" OR "lipid-based coatings" OR "composite coatings" or "antimicrobials" OR "natural antioxidants" OR "nutraceuticals" OR "colorants" OR "flavor agents" OR "nutrients" OR "plasticizers" OR "emulsifiers" and "antibrowning agents" OR "plant extracts" OR "essential oil" OR "synthetic antioxidants" OR "pH control agents" OR "moisture barrier" OR "gas permeability" OR "antimicrobial activity" OR "antioxidant activity" OR "mechanical properties" OR "sensory properties" OR "physiological responses" OR "physical properties" OR "thermal properties" OR "solvent casting" OR "emulsion preparation" OR "suspension" OR "hydrocolloid dispersion" OR "hot melt extrusion" OR "spray-drying" OR "nanotechnology" OR "biopolymer dissolution" OR "electrospinning" OR "decay" OR "weight loss" OR "texture changes" OR "colour change" OR "postharvest disease" OR "spoilage" OR "perishable" OR "physical damage" OR "microbial growth" OR "storage" OR "respiration" OR "temperature management" OR "humidity control" OR "ethylene sensitivity" OR "pests and pathogens" OR "packaging and storage" OR "transportation challenges" OR "market access and distribution" OR "waste and losses" OR "quality maintenance" OR "modified atmosphere packaging" OR "controlled atmosphere storage" OR "cold storage" OR "ethylene inhibitors" OR "ripening inhibitors" OR "smart packaging" OR "shelf life" OR "quality retention" OR "texture preservation" OR "colour stability" OR "microbial load" OR "flavour retention" OR "moisture sensitivity" OR "sensory acceptability" OR "surface roughness" OR "coating porosity" OR "adhesion strength" OR "barrier properties" OR "water vapor permeability" OR "gas permeability" OR "mechanical strength" OR "postharvest storage" OR "coating thickness" OR "coating adhesion" OR "shrivel" OR "decay" OR "physicochemical properties" OR "nutritional analysis" OR "edible coating systems implementation" OR "chemical analysis" OR "biodegradable" OR "environmental impact" OR "sustainable packaging" OR "biodegradable materials" OR "eco-friendly coatings" OR "green packaging" OR "reduced food waste" OR "reduced plastic use" OR "carbon footprint" OR "waste reduction" OR "compostable coatings" OR "low environmental impact" OR "reduced packaging waste" OR "natural ingredients" OR "green technology" OR "biodiversity conservation" OR "eco-packaging solutions" OR "food safety" OR "natural preservatives" OR "postharvest losses" OR "food waste reduction" OR "food security" OR "toxicity" OR "senescence" OR "metabolic changes" OR "nutrient retention" OR "respiration" OR "ethylene production and sensitivity" OR "moisture loss" OR "ripening management" OR "co-extrusion" OR "electrostatic coating" OR "roller coating" OR "immersion spinner" OR "misting" OR "microwave heating" OR "ultrasonic atomization" OR "dipping" OR "spraying" OR "brushing" OR "vacuum infusion" OR "layer-by-layer assembly" OR "smart coatings" OR "cost-effectiveness" OR "market acceptance" OR "consumer preferences" OR "circular economy" OR "economic viability" OR "nanotechnology" OR "encapsulation" OR "emulsions" OR "scaling up" OR "FDA regulations" OR "EFSA regulations" OR "food coating materials" OR "food contact substances" OR "codex alimentarius" OR "food safety regulations" OR "food additive regulations" OR "GRAS (generally recognized as safe) substances" OR "labeling requirements"

OR "food packaging regulations" OR "toxicological assessment" OR "novel food approval" OR "maximum residue limits" OR "food contact notification" OR "risk assessment" OR "compliance standards" OR "ingredient approval")].

Table S1: Most impactful academic journals for edible coatings and films for preserving fruit from 1998 to 2023 in Scopus database.

Sources	h_index	g_index	m_index	TC	NP	YFP
Postharvest Biology and Technology	20	21	1	2760	21	2004
LWT	18	21	1.385	1782	21	2011
International Journal of Biological Macromolecules	13	22	1.182	697	22	2013
Journal of Food Science	13	13	0.591	1094	13	2002
Food Chemistry	12	12	1.333	642	12	2015
Journal of Food Processing and Preservation	12	16	0.75	327	16	2008
Food And Bioprocess Technology	10	10	0.625	497	10	2008
Scientia Horticulturae	10	11	1.111	503	11	2015
Journal of Food Science and Technology	9	11	0.9	269	11	2014
Coatings	8	13	1.143	173	13	2017
Food Hydrocolloids	8	9	0.8	727	9	2014
Foods	8	11	2	218	11	2020
Food Packaging and Shelf Life	7	7	0.778	498	7	2015
Journal Of Agricultural and Food Chemistry	7	7	0.28	748	7	1999
Innovative Food Science and Emerging Technologies	6	7	0.462	350	7	2011
International Journal of Food Science and Technology	6	8	0.462	324	8	2011
Journal of Food Measurement and Characterization	6	9	1	119	9	2018
Journal of the Science of Food and Agriculture	6	6	0.231	217	6	1998
International Journal of Food Microbiology	5	5	0.313	462	5	2008
Polymers	5	8	0.833	318	8	2018

TC: Total Citations; NP: Number of publications; YFP: Year of the first publication

Table S2: Biomaterials used for forming edible coatings and films based on the documents published in the Scopus database before October 2023

Materials	NP	Materials	NP
Chitosan	136	Whey protein	4
Starch	62	Xanthan gum	4
Pectin	33	Sodium caseinate	3
Alginate	29	Gum cordia	3
Gelatin	24	Casein	3
Aloe vera	21	Tragacanth gum	2
Sodium Alginate	20	Cellulose nanocrystal	2
Carboxymethyl cellulose	18	Hydrocolloid	2
Hydroxypropyl methylcellulose	15	Seed gum	2
Mucilage	13	Quinoa protein	2
Gum arabic	13	Locust bean gum	2
Guar gum	10	Wheat gluten	2
Beeswax	10	Mastic gum	1
Apple	7	Flaxseed polysaccharides	1
Konjac glucomannan	6	Tara gum	1
Zein	6	Apricot gum	1
Pullulan	6	Banana puree film	1
Carrageenan	6	Papaya puree	1
Carnauba wax	5	Tomato	1

NP: Number of publications

Table S3: List of documents with ≥100 citations

Rank	TC	First author	Journals	Title of document	Research focus
1	405	Han et al.[38]	Postharvest Biology and Technology	Edible coatings to improve storability and enhance nutritional value of fresh and frozen strawberries (Fragaria × ananassa) and raspberries (Rubus ideaus)	Reducing decay and weight loss and enhancing shelf life and quality of fresh strawberries and raspberries in cold storage using chitosan-based coatings enriched with calcium and Vitamin E
2	350	Rojas-graü et al.[32]	Postharvest Biology and Technology	Apple puree-alginate edible coating as carrier of antimicrobial agents to prolong shelf-life of fresh-cut apples	Apple puree-alginate edible coatings with essential oils inhibit the growth of bacteria, yeasts, and molds, extending the shelf life of fresh-cut apples. Vanillin coatings were the most effective
3	306	Ponce et al.[39]	Postharvest Biology and Technology	Antimicrobial and antioxidant activities of edible coatings enriched with natural plant extracts: In vitro and in vivo studies	Chitosan coatings enriched with rosemary and olive enhanced antioxidant properties protecting the minimally processed squash of butternuts against browning reactions of squash. However, they did not present effective antimicrobial activity.
4	275	Velickova et al.[12]	LWT	Impact of chitosan-beeswax edible coatings on the quality of fresh strawberries (Fragaria ananassa cv Camarosa) under commercial storage conditions	Composite chitosan-based coatings prolonged the shelf life of strawberries in commercial storage conditions. The visual appearance, taste, and overall acceptance. A monolayer and three-layer technologies were used for coatings application.
5	271	Salvia-trujillo et al.[42]	Postharvest Biology and Technology	Use of antimicrobial nanoemulsions as edible coatings: Impact on safety and quality attributes of fresh-cut Fuji apples	Nanoemulsion-based edible coatings containing 0.1% (v/v) essential oil presented higher E. coli inactivation and slower psychrophilic bacteria growth on fresh-cut apples than conventional emulsions during storage time.
6	269	Raybaudi-massilia et al., 2008	International Journal of Food Microbiology	Edible alginate-based coating as carrier of antimicrobials to improve shelf-life and safety of fresh-cut melon	Composite alginate-based edible coating (malic and essential oils) improved physicochemical and sensory quality of the fresh-cut melon. It also prolonged their shelf life and safety by inhibiting native flora growth and *Salmonella enteritidis*.
7	261	Otoni et al., 2014	Food Hydrocolloids	Antimicrobial and physical-mechanical properties of pectin/papaya puree/cinnamaldehyde nanoemulsion edible composite films	The study showed that adding into the films of papaya puree increased its extensibility and water vapour permeability and that of cinnamaldehyde inhibited *E. coli, S. enterica, L. monocytogenes*, and *S. aureus*.
8	222	Vu et al.[78]	Food research International	Development of edible bioactive coating based on modified chitosan for increasing the shelf life of strawberries	Edible bioactive coatings based on modified chitosan have been formulated to control moulds and total flora present on strawberries and prolong their shelf life during cold storage (4 °C). Red thyme and oregano essential oils, limonene, and peppermint were applied as bioactive agents.
9	206	Tanada-palmu et al.[79]	Postharvest Biology and Technology	Effect of edible wheat gluten-based films and coatings on refrigerated strawberry (Fragaria ananassa) quality	The potential of edible wheat gluten-based films and coatings to improve shelf life and maintain the quality of strawberries in refrigerated conditions has been investigated. The results indicated that the bilayer application of

					the coatings reduced firmness and weight losses and maintained the visual quality of the fruit during the storage time.
10	194	Rojas-graü et al.[80]	Journal of Agricultural and Food Chemistry	Mechanical, Barrier, and Antimicrobial Properties of Apple Puree Edible Films Containing Plant Essential Oils	Barrier, mechanical and antimicrobial properties of apple puree-based edible films incorporated by essential oils have been studied. Adding essential oils (oregano, cinnamon, lemongrass) into films improved their effectiveness against *E. coli*, maintained tensile properties and altered film permeability
11	187	Basiak et al.[81]	Polymers	How Glycerol and Water Contents Affect the Structural and Functional Properties of Starch-Based Edible Films	The addition of glycerol improved the functional properties (solubility and water absorption) of the Strach-based edible films and reduced slightly (not significant) mechanical properties (tensile strength at break and Young's modulus)
12	178	Brasil et al.[9]	LWT	Polysaccharide-based multilayered antimicrobial edible coating enhances the quality of fresh-cut papaya	Microencapsulated beta-cyclodextrin and trans-cinnamaldehyde complex in multilayered edible coating based on chitosan and pectin improved coatings antimicrobial properties; reduced firmness, vitamin C and carotenoids losses, and maintained colour and juice leakage of fresh-cut papaya. It also extended the shelf life of fresh-cut papaya up to 15 days at 4 °C
13	176	Genskowsky et al.[82]	LWT	Assessment of antibacterial and antioxidant properties of chitosan edible films incorporated with maqui berry (Aristotelia chilensis)	The study showed that chitosan-based edible films enriched with maqui berry extracts present significant antioxidant and antibacterial properties able to delay microbial growth and improve the oxidative stability of packed foods.
14	166	Bill et al.[83]	Crop Protection	The efficacy of combined application of edible coatings and thyme oil in inducing resistance components in avocado (Persea americana Mill.) against anthracnose during post-harvest storage	This paper indicated that Chitosan-based combined with thyme oil (3:1 v/v) induced a significant resistance component in avocados against anthracnose disease.
15	158	Maqbool et al.[41]	Journal of Agricultural and Food Chemistry	Effect of a novel edible composite coating based on gum arabic and chitosan on biochemical and physiological responses of banana fruits during cold storage	The paper showed that Gum (10%) and chitosan (1%) composite coating reduced the weight, total carbohydrates and firmness losses of bananas extending their shelf life up to 33 days in commercial handling conditions
16	144	Du et al.[84]	Journal of Food Science-a	Effects of allspice, cinnamon, and clove bud essential oils in edible apple films on physical properties and antimicrobial activities	The physical and antimicrobial properties of apple puree-based films incorporated with essential oils (allspice, cinnamon, and clove bud) have been investigated. Results showed that oil incorporation increased film effectiveness against foodborne pathogens (*E. coli O157:H7*, *S. enterica,* and *L. monocytogenes*) without affecting its physical properties (particularly water vapour permeability)

17	140	Orsuwan et al.[85]	Food Hydrocolloids	Preparation of antimicrobial agar/banana powder blend films reinforced with silver nanoparticles	The paper presented the preparation of a binary blend film/coatings (agar/banana powder) and their composite films enriched with silver nanoparticles using the casting method. The physicochemical and functional properties of A/B blend films improved with banana powder concentration while mechanical properties decreased. The composite film exhibited a high antibacterial activity against foodborne pathogenic bacteria.
18	137	Mantilla et al.[10]	LWT	Multilayered antimicrobial edible coating and its effect on quality and shelf-life of fresh-cut pineapple (Ananas comosus)	Multilayered alginate-based edible coating with microencapsulated antimicrobial complex was assessed for its ability to improve the quality and shelf life of fresh-cut pineapple stored at 4 °C. The coating exhibited significant activity against psychotropics and yeast and molds, preserved juice leakage and texture and did not affect the sensory quality of fresh-cut pineapple whilst prolonging shelf life by up to 15 days.
19	135	Nawab et al.[86]	International Journal of Biological Macromolecules	Mango kernel starch as a novel edible coating for enhancing shelf- life of tomato (Solanum lycopersicum) fruit	Edible coatings based on mango kernel starch prepared with different plasticizers were evaluated in prolonging tomato shelf life. The coating containing sorbitol has been most effective in maintaining tomato quality (physicochemical and sensory) by delaying the ripening process and prolonging shelf life up to 20 days at 20 °C.
20	134	Ali et al.[87]	International Journal of Biological Macromolecules	Starch-based antimicrobial films functionalized by pomegranate peel	Starch-based films have been functionalized by encapsulating with pomegranate peel extracts. Adding pomegranate peel extracts inhibited bacteria growth (*Salmonella* and *S. aureus*) and reinforced the film's properties by increasing Young's modulus, tensile strength, and stiffness.
21	134	Mohammadi et al.[88]	Postharvest Biology and Technology	Chitosan nanoparticles loaded with Cinnamomum zeylanicum essential oil enhance the shelf life of cucumber during cold storage	This study showed that the encapsulation of Cinnamomum zeylanicum essential oil (1.5g/L) into chitosan nanoparticles (CSN)-based coatings significantly decreased both disease severity and incidence of *Phytophthora drechsleri*-inoculated cucumbers stored during 7 d at 4°C followed by additional days (2-3) at 20°C.
22	132	Park et al.[89]	Journal of Food Science	Antifungal Coatings on Fresh Strawberries (Fragaria × ananassa) to Control Mold Growth During Cold Storage	Chitosan-edible coatings have been evaluated in controlling mould growth on fresh strawberries. The chitosan-based coating has been effective against *Cladosporium* sp. and Rhizopus sp. whereas the combination with Potassium sorbate did not have significant antifungal activity.
23	129	Choi et al.[65]	LWT	Characterization of edible film containing essential oils in hydroxypropyl methylcellulose and its effect on quality attributes of 'Formosa' plum (Prunus salicina L.)	Hydroxypropyl methylcellulose (H) films prepared by incorporating essential oils were characterised and evaluated in preserving fresh plums. 2%H films with oregano essential oil (2%) significantly delayed respiration, ethylene production, weight loss, fruit softening and microbial growth in stored plums.

24	128	Kaya et al.[40]	Innovative Food Science & Emerging Technologies	Production and characterization of chitosan based edible films from Berberis crataegina's fruit extract and seed oil	The paper investigated chitosan-based edible films supplemented with *Berberis crataegina* seed oil and fruit extract. Chitosan-fruit extract film presented the highest thermal stability, antioxidant, antimicrobial and antiquorum activity while Chitosan-*B. crataegina*'s seed oil showed the highest hydrophobicity.
25	126	Sipahi et al.[90]	LWT	Improved multilayered antimicrobial alginate-based edible coating extends the shelf life of fresh-cut watermelon (Citrullus lanatus)	The effect of antimicrobial alginate-based edible coating applied using a layer-by-layer technique on fresh-cut watermelon has been studied. The coating enhanced sample texture and consumer acceptance. The application of multilayered edible coating with 1 or 2g/100g alginate prolonged the shelf life of fresh-cut watermelon from 7 (control) to 12–15 days.
26	122	Tesfay et al.[91]	Food Packaging and Shelf Life	Evaluating the efficacy of moringa leaf extract, chitosan and carboxymethyl cellulose as edible coatings for enhancing quality and extending postharvest life of avocado (Persea americana Mill.) fruit	The study presented the effectiveness of carboxymethyl cellulose and chitosan-based coatings with moringa leaf extract in avocado storage. The combination of moringa leaf extract with edible coatings improved the phytochemical characteristics of avocado and prolonged its shelf life by reducing the mass loss
27	121	Du et al.[92]	Journal of Agricultural and Food Chemistry	Storage Stability and Antibacterial Activity against Escherichia coli O157:H7 of Carvacrol in Edible Apple Films Made by Two Different Casting Methods	This paper showed that the apple-based edible films containing carvacrol stored at 5 °C and 25 °C for 49 days had high antimicrobial activity and low water vapour and oxygen permeability.
28	117	Maqbool et al.[93]	Crop Protection	Control of postharvest anthracnose of banana using a new edible composite coating	The paper showed that the composite coating (10% Arabic gum and 1% chitosan) is more effective against banana anthracnose than individual ingredient coatings. It effectively delayed the ripening process of bananas during storage.
29	115	Medina et al.[94]	Journal of Food Engineering	Chitosan thymol nanoparticles improve the antimicrobial effect and the water vapour barrier of chitosan-quinoa protein films	Research showed that chitosan-based films with thymol nanoparticles had high antimicrobial activity against B. cinerea. The addition of nanoparticles into films reduced their water vapour permeability and improved blueberries and tomatoes' shelf life.
30	112	Arroyo et al.[95]	Food Chemistry	Antimicrobial active edible coating of alginate and chitosan add ZnO nanoparticles applied in guavas (Psidium guajava L.)	Chitosan, alginate, and composite (chitosan and alginate) coatings incorporating nanoZnO presented effective antibacterial activity. Chitosan-based coating and composite coating were more effective against physicochemical changes related to maturation prolonging guava shelf life up to 20 days versus 7 days for control fruits.
31	111	De Aquino et al.[96]	Food Chemistry	Impact of edible chitosan–cassava starch coatings enriched with *Lippia gracilis* Schauer genotype mixtures on the shelf life	Composite coatings (chitosan (CH) and cassava starch (CS)) enriched with a mixture of *lippia gracilis* Schauer genotypes essential oil (EOM) has been investigated. The composite coatings (2%CH-2%CS-EOM (1, 2, 3%) were most effective in inhibiting bacteria growth on guava extending its shelf life

				of guavas (Psidium guajava L.) during storage at room temperature	
32	109	Ali et al.[31]	Food Packaging and Shelf Life	Antimicrobial activity of chitosan enriched with lemongrass oil against anthracnose of bell pepper	Combining 1%chitosan-based coating with lemongrass essential oil (0.5%) was most effective in controlling microbial growth on bell peppers and maintaining bell pepper quality.
33	108	Martiñon et al.[97]	LWT	Development of a multilayered antimicrobial edible coating for shelf-life extension of fresh-cut cantaloupe (Cucumis melo L.) stored at 4 °C	The composite edible coating (2% chitosan + 1% pectin + 2% trans-cinnamaldehyde) prepared using the layer-by-layer technique is most effective in inhibiting microbial growth on fresh-cut cantaloupe, in maintaining physicochemical and sensory attributes prolonging shelf life (7-9 days) versus 4 days for controls)
34	107	Fagundes et al.[98]	Postharvest Biology and Technology	Effect of antifungal hydroxypropyl methylcellulose-beeswax edible coatings on gray mold development and quality attributes of cold-stored cherry tomato fruit	Edible composite coatings based on hydroxypropyl methylcellulose (HPMC) and beeswax (BW) combined with additives reduced gray mold development on cherry tomatoes. However, only combination with ammonium carbonate (AC) controlled weight loss and maintained firmness.
35	107	Martelli et al.[99]	Journal of Food Science	Effect of chitosan nanoparticles and pectin content on mechanical properties and water vapour permeability of banana puree films	The addition of chitosan particles in banana puree-based edible films significantly reduced water vapour permeation and adding pectin (0.5%) and glycerol (5%) Significantly improved film elongation implying better plasticity and handablility.
36	105	Saberi et al.[100]	Postharvest Biology and Technology	Application of biocomposite edible coatings based on pea starch and guar gum on quality, storability, and shelf life of 'Valencia' oranges	Incorporating lipid compounds into bio-composite edible coatings based on pea starch and guar gum reduced respiration, weight and firmness loss and decay of oranges during storage (4 weeks at 5 °C + one week at 20 °C). Its application using the single-layer technique recorded greater scores for overall flavour and freshness than uncoated or its application by layer-by-layer technique.
37	102	Du et al.[101]	Journal of Food Science	Antibacterial Effects of Allspice, Garlic, and Oregano Essential Oils in Tomato Films Determined by Overlay and Vapor-Phase Methods	Tomato puree film encapsulated with essential oils (oregano, allspice, garlic) presented a strong antimicrobial activity against *E. coli*, *S. enterica*, and *L. monocytogenes*. The EOs increased elongation and darkened the colour of tomato-based antimicrobial films without affecting water vapour permeability.
38	100	Treviño-garza et al.[102]	Journal of Food Science	Edible Active Coatings Based on Pectin, Pullulan, and Chitosan Increase Quality and Shelf Life of Strawberries (Fragaria ananassa)	Edible active coatings (EACs) based on pectin, pullulan, and chitosan incorporated with sodium benzoate and potassium sorbate reduced weight loss and colour alteration, and improved sensory quality (colour, flavour, texture, and acceptance) of strawberries. they also reduced microbial growth (total aerobic counts, molds, and yeasts) in strawberries
